# On the Suitability and Potential of Nursing Care Discussion Forums as a Health Promotion Measure for Long-Distance Caregiving Relatives: Evidence from Upper Austria

**DOI:** 10.3390/healthcare7040139

**Published:** 2019-11-07

**Authors:** Tatjana Fischer, Markus Jobst

**Affiliations:** 1 Institute of Spatial Planning, Environmental Planning and Land Rearrangement, University of Natural Resources and Life Sciences Vienna, Peter-Jordan-Straße 82, 1190 Vienna, Austria; 2 Department for Geodesy and Geoinformation, Research Group Cartography, Vienna University of Technology, Erzherzog-Johann-Platz 1/120-6, 1040 Vienna, Austria; markus@jobstmedia.at

**Keywords:** long-distance caregiving relatives, health promotion, nursing care discussion forums, requirements, problems of fit, location planning, spatial context, expert survey, Upper Austria

## Abstract

*Background*: The number of persons who have to overcome extensive geographical distances for caring for their older parent(s), hereinafter referred to as long-distance caregiving relatives (LDCs), is rising. However, in the non-English-speaking Global North, little is known about the LDCs’ health literacy and the design of tailor-made health promotion measures for this target-group. Using the example of nursing care discussion forums (NCDF), this paper reflects the requirements and (future) potential of professionally-lead support groups for LDCs on the case-study example of Upper Austria. *Methods*: In order to approach this unexplored topic considering spatial-related aspects, a qualitative-explorative study design was chosen, focusing on the providers’ perspective. A written survey among all NCDF-group leaders was carried out. *Results*: LDCs do not make use of NCDFs at present. It is considered that this is above all for time constraints, lack of information and location-based problems of fit. This applies for urban as well as rural contexts. *Conclusions*: LDCs need more attention in public health. Suitable NCDFs have to be located in the LCDs’ residential municipalities and have to fulfill different requirements from those of local caregiving relatives, particularly with regard to purpose and scope.

## 1. Introduction

Due to demographic aging—caused by an increase in life expectancy and declining birth rates [1,2]—the discussion on financing care services for the elderly and the fact that nursing careers differently takes long [3], especially in the Global North, family caregiving relatives have become a target group of public health [4,5]. This is because caregiving relatives are an essential pillar of domestic care for older family members [5,6] and help to fulfill the central wish of older people—namely, staying at home as long as possible [7]. Providing (informal) domestic care is linked with physical strains as well as psychological strains and affects both the caregiver’s well-being and—in the long run—health [8,9,10,11,12,13,14]. The impacts on health and well-being vary individually, because caregiving related overloads and burdens are linked to personality such as the degree of emotional involvement and ability to distance oneself from expectations of others, coping strategies and health literacy, the life-cycle position related to employment and child-care, as well as the amount of accompanying support related to domestic care for the elder family member(s).

In order to support caregiving relatives, over time, a number of support and relief measures have been developed. These health promotion measures comprise a variety of information and counseling services for dealing with the care situation and finding external (professional) support [4,5,11,15]. Among them and in comparison to other relief measures such as holiday offers for informal caregivers and offers for replacement care, are Austria nursing care discussion forums (NCDFs), which have a long tradition and serves as short breaks from caregiving [5] and primarily address local caregiving relatives who are either living together with the cared-for person under a single roof or next to the cared-for person [5].

Thus far, the efficiency of professionally-led support groups or NCDFs and their added value for psychological well-being and health of caregivers of cared-for elder persons of different clinical pictures is discussed controversially [16,17,18,19,20,21,22], while the issue of demand and location planning as well as the design of health promotion and public health measures—in comparison to local caregiving relatives [14,23,24,25,26,27,28]—are disregarded. It is precisely the latter aspect that needs attention regarding a considerable proportion of informal caregivers whose amount will increase in the future [25,29], namely long-distance caregivers. This also applies for their coping strategies, health literacy and use of health promotion measures [24,25].

### Aim of the Paper and Research Questions

In order to reach the United Nations Sustainable Development Goals’ overarching aim to leave no one behind and in order to preserve good health and well-being to all regardless of gender, age and place of residence according to the Shanghai Declaration 2016 [30], this paper deals in the context of target-group appropriate social infrastructure planning with the requirements and potential of nursing care discussion forums as a health promotion measures for LDCs. The spatial reference framework is Upper Austria, a province of Austria. Austria is a high-income member state of the European Union, but faces an increasing polarization between rapidly growing urban areas on the one hand and aging rural or alpine peripheries on the other hand [31,32]. LDCs are a neglected target group in public health and sound empirical evidence on health literacy and making use of existing health promotion measures including NCDFs is missing [33,34].

This paper addresses LDCs who belong to the second generation of caregiving relatives, namely daughters and sons as well as daughters-in-law and sons-in-law. The reason for this scoping lies in the fact of an increasing imbalance related to the distribution and age-structure of the population, the trends towards the beanpole family [35] and the spatially differentiated potential of family care due to (Upper) Austria’s growing spatial heterogeneity between (infra-)structurally strong and weak areas [34,36]. These factors are strongly linked and have already led to a decrease of supportive caregiving members within the families themselves on the one hand and a growing number of LDCs in Austria [34] on the other hand. In turn, it can be assumed that this already may have led and still will lead to the LDCs’ increased focus on care for their parent(s).

Taking into account the LDCs’-specific burdens and constraints, three research questions are posed, in order to assess the present offer and if necessary, to derive prerequisites to raise the NCDFs’ suitability:

1. What do we know about the LDCs’ attendance of NCDFs in different spatial settings?
2. What kinds of (spatial-related) factors determine the LDCs’ use of NCDFs?
3. Do present NCDFs meet the requirements of LDCs and what can be done to increase the attractiveness of this health promotion measure for this target group?

## 2. Literature Review: Nursing Care Discussion Forums and Long-Distance Caregiving

### 2.1. Nursing Care Discussion Forums as a Support Measure for Informal Caregivers

For this paper nursing care discussion forums (NCDFs) are defined as a type of support group for informal caregivers of (older) people in need of care in order to maintain or enhance the caregivers’ mindfulness—a focal component of psychological health [37]—well-being and stress coping capacity [18].

NCDFs take place in secure surroundings, are led by nursing or health care professionals and offer space for mutual exchange among caregivers, provide emotional support, information and counseling [16] and require experienced and highly-skilled group leaders in order to be able to sufficiently deal with particular caregiving situations [18]. Moreover, NCDFs helps to build communities among caregivers [16].

It was found that non-mixed NCDFs related to the clinical picture of the cared-for (older) person of the participants [ibid] and a small group size [18] are relevant factors for success of this intervening support measure. For this reason, over the years a range of NCDFs came into existence, above all focusing on informal caregivers of persons who suffer from Alzheimer and dementia [18,19].

### 2.2. Long-Distance Caregiving Family Members—A Particular Target Group in Health Promotion

Caregiving relatives are a heterogeneous group related to availability for caregiving reasons and the spatial constellation related to the places of residence of the caregivers on the one hand and the cared-for-family member(s).

Spatially spoken, caregiving relatives can be split into two groups: local caregivers and distance caregivers. The latter mostly belong to the second generation of carers (daughters, sons) [14,35] and among them, there is a subgroup called long-distance caregivers (LDCs), who have a time expose of at least one hour in order to overcome a geographical distance of at least 50 miles (80 kilometers) between their place of residence and those of their cared-for parent(s) [38,39].

Due to an ongoing spatial concentration of higher education facilities and labor markets in urban agglomerations and mostly rural-urban directed internal and international migration flows [27,40], as well as due to retirement migration [26], overcoming (extensive) distances for caregiving reasons has become a widespread phenomenon internationally and overcoming extensive geographical distances is an obstacle for those relatives who are willing to engage (more) in domestic care for their parent(s) [1,24,29,41]. International research on LDCs—mainly from the US—shows that the necessity to overcome extensive geographical distances impacts the intensity as well as the kind of support or care for the older parent(s), but not the strength of emotional bonds [ibid]. Long-distance caregiving results in different kinds of involvement related to the care situation itself [26]. LDCs take on different tasks such as coordination and organization of professional domestic care, financial donations, provide support for those caring family members who bear the main burden of care [23,39,40,41,42].

LDCs suffer from completely different challenges compared to local caregivers [26,29]. These include: time exposure and financial expenses of (regularly) commuting between at least two places of residence, being torn between perceived obligation, the desire to give something back and the actual opportunity to engage in domestic care, as well as living with uncertainties related to information on the actual status of well-being and health of the older parent(s), being condemned to observe the older parent(s) physical and mental deterioration from afar and experience conflicts with other family members who provide most of the care locally [24,25,26,29,39,43].

Life and stress situations of LDCs individually differ and depend on the degree of care need, the clinical picture, particularly cancer and dementia, which are becoming more and more relevant [23,42,44], the availability of formal and informal support on-site [45] and the LDCs’ attitude or readiness to commute, as well as the geographical distance itself [25,46]. In addition, it was found that women tend to be more willing to overcome longer geographical distances for caregiving reasons than men [1]. It can be assumed that the care situation is particularly stressful for middle-aged female LDCs.

Nevertheless, there is a wide range of support or health promotion measures for LDCs [28] covering comprising on-site or face-to-face support measures (e.g., peer support) and web-based measures.

Since it has been shown that the availability of direct contact to the cared-for person at any time has a calming effect to LDCs and promotes their well-being [46], a variety of web-based support measures has been developed over the years, focusing primarily on bridging geographical distance associated with communication between LDCs and the cared-for person (e.g., by means of video telephony [40] and virtual exchange between LDC, the cared-for person and formal caregivers [23]). All these web-based solutions are advertised on relevant internet platforms.

Additionally, there are a number of online-guides addressing amongst others two core issues: (1) providing effective care from distance [47] and (2) the presentation of coping strategies for dealing with this challenging situation [48,49]. They contain various advice for dealing with care at a distance and to provide an overview of other contact addresses and web links.

## 3. Materials and Methods

The present paper deals with a new topic in public health in Austria, and because of its complexity, a mixed-methods-approach was applied [50]. The selected information sources were as follows:

- An existing dataset that originates from an explorative-qualitative written survey of group leaders of the Upper Austrian NCFDs conducted in 2017.
- Secondary and statistical data comprising (1) a summary list of all NCDFs in Upper Austrian NCDFs, including information on location municipalities, provided by the provincial administration of Upper Austria, (2) statistical data on care allowance recipients in Upper Austria, provided by the provincial administration of Upper Austria and (3) the official dataset on the spatial types of the Austrian municipalities, namely the Urban-Rural-Typology of the Austrian municipalities, provided by Statistics Austria [51].

This section describes the information sources including reasons for selection, procedure of data collection, data analysis and triangulation.

### 3.1. Information Sources, Handling of Data and Data Analysis

#### 3.1.1. Information Source 1: Results of a Written Survey of Group Leaders of the Upper Austrian NCDFs Conducted in 2017

In summer 2017, the first author of this paper received an invitation to contribute to the anthology “Fair opportunities for healthy aging” on the occasion of the 20th anniversary of the Fund for a Healthy Austria. The initial consideration was to draft a conceptual contribution on LDCs in Austria in order to introduce this target group to the relevant stakeholder in public health. At the same time, an employee of the provincial administration of Upper Austria—also invited to contribute—was interested in writing an article about NCDFs and their relevance for caregivers in Upper Austria. Within the scope of a telephone conversation, both ideas were linked. Due to the fact that in Austria LCDs have not yet been explored before, it was decided to conduct a cross-sectional qualitative-explorative written survey with the group leaders of the Upper Austrian NCDFs in order to understand the providers’ perspective referring to the interrelations of demand, supply and location issues that determine the use of NCDFs of two different groups of caregiving relatives: local caregivers and LDCs. It turned out that a target group-specific analysis went beyond the editor’s scope of the publication. With regard to LDCs, the joint publication [33] concluded by pointing out the necessity to fathoming the relevance of NCDFs as a health promotion measure for LDCs.

In the following, the procedure of data collection and data analysis of the dataset are described; intending to collect as much information as possible on LDCs, it was aimed to attract as many NCDF-group leaders as possible for the written survey. The employee of the Upper Austrian provincial administration—second author of the above mentioned publication [ibid]—who is professionally responsible for the supervision of the Upper Austrian NCDFs and over the years has stayed in good contact with the NCDF-group leaders, invited all of them (there were 77 Upper Austrian NCDFs as of 2017) by mail to participate in a written survey.

Due to the lack of literature on the service-specific suitability of NCDFs in the context of long-distance caregiving and the lack of information on LDCs in the Austrian context in general as well as on the concrete schedule of NCDFs and on how the NCDF-group leaders document each session, a theoretically-led formulation of categories for closed-questions seemed to be inappropriate. In order to provide the opportunity to respond narratively and spontaneously, it was decided for mostly open-ended questions [51] addressing target group-specific (local caregiving relatives as well as LDCs) issues as follows:

- The actual catchment area of the NCDFs.
- The target group-specific (LDCs’) actual demand.
- Ideas on ideal health promotion for LDCs.
- The suitability of NCDFs for LDCs.

A questionnaire consisting of 25 questions (see Table 1) was prepared by the first author of this paper and the employee of the Upper Austrian provincial administration. The latter also took on the task of validation of the questionnaire related to content and wording due to her educational background in nursing and responsibility for the quality assurance of the Upper Austrian NCDFs.

In fall 2017, in a second e-mail, the questionnaire—attached in the form of a Word document—was sent to the NCDF group leaders. They were asked to insert the questions directly into the Word document and to send it back within a period of three weeks. In a cover letter written by the employee of the Upper Austrian provincial administration, it was pointed out that non-participation was fully respected. Since the written survey was conducted with official permission of the appropriate department of the Upper Austrian provincial administration and did not address investigative medical research, no ethic approval from an ethics committee was required. Nonetheless, compliance with all international research standards—above all, warranting voluntary participation without fear of negative consequences in case of refusal; and confidential handling of data—was met.

By the end of November 2017, 47 out of 77 NCDF-group leaders (61%) took part in the survey and submitted the completed questionnaires by e-mail, mail or fax.

#### 3.1.2. Information Source 2: Summary List of Upper Austrian NCDFs

This summary list in form of a Word document was provided by the Upper Austrian provincial administration. It contained current information on the number and the catchment areas of the NCDFs (as of 2017). Two different types of NCDFs can be distinguished: (1) NCDFs with a catchment area that is limited to the location municipality, hereafter referred to as eponymous municipalities, and (2) NCDFs with regional catchment, namely the eponymous municipality and the participating partner municipalities, hereafter referred to as nursing care discussion clusters.

#### 3.1.3. Information Source 3: Urban-Rural-Typology of Statistics Austria

All municipalities where NCDFs were located were categorized, using the Urban-Rural Typology of Statistics Austria [52], which offered 10 different spatial categories based on several indicators, such as the number of inhabitants, commuter relationships between municipalities, reachability of urban centers and the level of infrastructure facilities. In order to be able to carry out a spatial-related analysis of the NCDFs, all Austrian municipalities were grouped into the following four spatial categories: urban centers, regional centers, suburban municipalities and rural municipalities.

### 3.2. Mapping the NCDFs’ Coverage and Catchment Areas

In order to map the coverage of the Upper Austria with NCDFs the information from the summary list were transferred to a table and merged with geographic information. The process of data wrangling precedes the integration of the NCDF and geoinformation. Whereas the NCDF table requested a manual harmonization of the indicator identifiers according to their way of writing and temporal validity, the requested geoinformation had to be extracted and validated for the proposed time of survey. Although all data are accessible in Austria, the given quality did not prevent from manual data wrangling.

In order to be able to estimate the representativeness of the response rate regarding to the NCDFs’ theoretical catchment referring to caregiving relatives of domestically cared-for older people, administrative data from Upper Austria on the recipients of care allowance were used. These data refer to the number of care allowance recipients at the municipal level, but are not classified according to age or sex of the recipients for reasons of data protection.

In order to determine the proportion of domestically cared-for older people among all care allowance recipients, the following assumptions were made: firstly, it was assumed that the share of older people made up 98%; secondly, 80% of these older people received care or support from their relatives.

### 3.3. Assessment of the Current NCDF-Offer for LDCs and Implications

**Figure 1:**
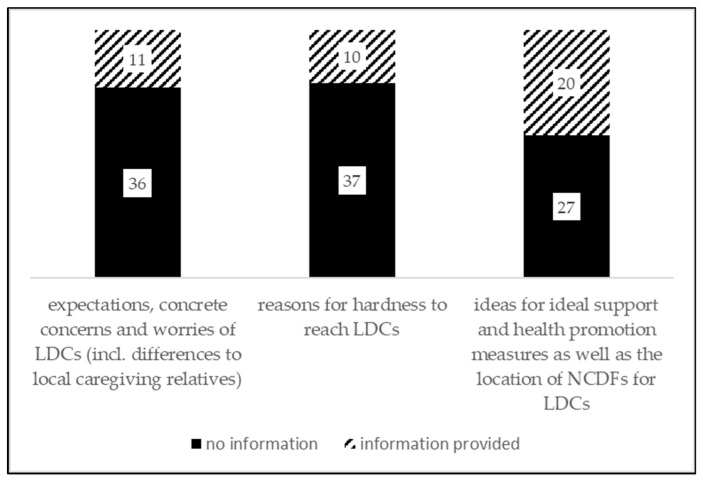
Available information on LDCs.

In a first step, the questionnaires were numbered chronologically according to their receipt. The registered answers—the quality of information referring to questions decidedly addressing LDCs (see Figure 1) ranged from a few notes to short sentences and paragraphs—were transferred verbatim to an Excel file (version 26 for Windows). The closed questions were coded in a binary form (0 = no/not appropriate, 1 = yes/appropriate; missing answers = 99).

In a second step, categories were developed according to the comparative method of Glaser and Strauss [53]. For this purpose, the information related to the open-ended questions were binary coded (0 = no/not appropriate, 1 = yes/appropriate; missing answers = 99), then transformed to an SPSS database (version 24 for Windows, IBM, Armonk, NY, USA).

In a third step, in order to assess the data, they were analyzed theme-centered according to Boyatzis [54].

The LCD-related responses (see Figure 1) formed the starting point for reflecting the demand, supply and location issues and further LCD-suitable NCDF-development. In order to be able to assess the suitability of the current NCDF-offer, the gathered information referring to LDCs was compared to those for local caregiving relatives.

On this basis, the LCD-suitability of the current NCDF-offer was considered including implications for necessary or possible improvement by comparing the NCDF-requirements for LDCs and local caregiving relatives.

As Figure 1 shows, LCD-related questions have been answered by comparatively few NCDF-group leaders: 21 out of 47 responding NCDF-group leaders at least have answered one out of three questions related to LDCs, four group leaders have answered all three questions. In order to reliably assess the Upper Austrian situation, it was decided to conduct a descriptive statistical analysis and to discuss the results—if possible and suitable—with (international) literature.

## 4. Results

**Figure 2:**
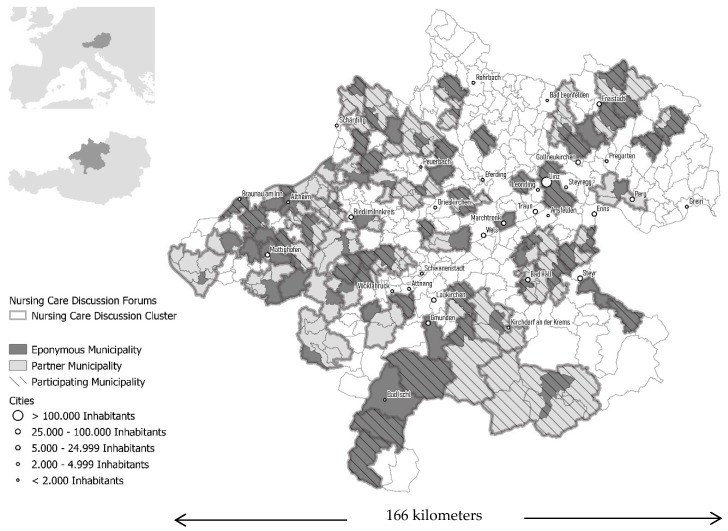
Spatial organization structure of the Upper Austrian NCDFs (as of 2017). The dark colored and bordered areas (referred to as eponymous municipalities) show NCDF-municipalities without further cooperation municipalities. The light gray colored municipalities (referred to as partner municipalities) together with the respective eponymous municipalities (=dark colored areas) constitute a nursing care discussion cluster (=regional NCDF-cooperation). The borders mark the delimitation of the nursing care discussion clusters which consist of neighboring municipalities. The hatched areas in turn, designate those NCDF-municipalities or nursing care discussion clusters which are covered by the written survey.

In 2017, NCDFs were located in 195 out of the then 442 Upper Austrian municipalities. It was found that:

- With the exception of NCDFs located in regional centers, all other spatial types of NCDF-municipalities were covered by the expert survey, namely 50 % of the urban NCDFs, 75% of the suburban NCDFs and 60% of the rural NCDFs (see Table 2).
- Out of all Upper Austrian municipalities, 57% were eponymous municipalities or partner municipalities, that built a Nursing Care Discussion Cluster (see Figure 2).
- The coverage related to the theoretical catchment area of the NCDFs referring to domestically cared-for older persons: 70% of the theoretical catchment could be recorded (see Figure 2).
- From the written expert survey, information on 61% out of all 77 Upper Austrian NCDFs could be obtained (see Table 2).

### 4.1. Result 1: Catchment and Reachabilityof NCDFs

NCDFs are health promotion measures located near to the places of residence of the participants and mostly are of local reach. Although being intensively advertised, only a small number of caregivers make use of this offer. The participants mostly live in the same municipality as the cared-for older person(s) or parent(s). Joining NCDFs apparently justifies a short break from caregiving obligations. There are no LDCs among the NCDF-participants.

#### 4.1.1. A Portrait of NCDFs

NCDFs are run by highly skilled health and nursing staff, especially women, and are places of personal, professional and confidential exchange of caregivers’ central concerns and worries. NCDFs are held in selected locations such as restaurants, suitable rooms in the municipal office as well as in nursing homes. Normally, NCDFs take place once or twice a month, usually during the week and in the evening [33] (see Table 3). The NCDF-group leaders receive a small expense allowance for this service.

#### 4.1.2. NCDFs’ Catchment Areas

The catchment areas of the NCDFs correspond to the places of residence of the caregivers. In none of these cases, the actual catchment area extends beyond the nursing care discussion cluster or cooperating neighbor municipalities. 29 out of 47 NCDFs are designed as nursing care discussion clusters comprising at least two neighboring municipalities in which the NCDFs are alternately held. In 26 out of 29 cases, one NCDF-group leader is responsible for all involved municipalities. The theoretical catchment area of the 47 NCDFs or nursing care discussion clusters comprises 82 municipalities, including about 18,700 domestically cared-for older people supported by family members. In contrast, the actual catchment area comprises 37 municipalities with about 15,000 domestically care-for older people who are cared for at home by family members.

The actual catchment area of the NCDFs covers 45% of the involved municipalities and 80% of the domestically cared-for older people who receive (care) support from relatives. Referring to the latter aspect, there are differences related to the spatial type of the eponymous NCDF-municipalities (see Table 4): this is just under 100% for NCFDs located in urban municipalities, 4% for the suburban and 56% for the rural NCDFs. This means that the deviations between the theoretical and the actual catchment area of rural NCDFs are greatest or rather that the catchment area of an NCDF is tended to be limited to the eponymous municipality.

#### 4.1.3. Profile of NCDF-Participants

Looking at the profile of the NCDF-participants, it can be seen that they belong to the first and second generation of caregiving relatives and are aged 45 to 70 years. The participants are mainly women supporting or caring for their parent(s) or parent(s)-in-law. The NCDF-participants live primarily in the same municipality as the cared-for older relative, except for one urban NCDF, which cannot be explained on the basis of the information available. LDCs cannot be found among the participants [33]. Moreover, NCDFs are relevant for a small number of caregivers and show a tendency to decline regarding their use [ibid] (see Table 5). With regard to these facts and to the sociodemographic profile of the NCDF-participants, the NCDFs in urban, suburban as well as in rural municipalities hardly differ from each other.

To sum up, NCDFs particularly are a health promotion measure for local caregiving relatives.

#### 4.1.4. Advertising Channels and Reachability of LDCs

Irrespective, of whether or not NCDFs are located in urban, suburban or rural municipalities, according to the NCDF-group leaders’ statements, NCDFs are advertised using various channels (see Figure 2 and Table 6). Overall, 20 different advertisement channels could be distilled from empirical data. These channels, in turn, can be clustered into four groups, taking into account the geographical scope and the way in which caregivers are contacted:

- Advertisement channels of local scope.
- Advertisement channels of regional scope.
- Directly addressing of caregiving relatives.
- Unspecified advertisement channels in terms of both, their (spatial) scope and contact building with caregiving relatives.

It turns out that the advertisement of the NCDFs primarily focuses on the local level. In total, NCDFs were advertised through 11 different advertisement channels, including:

- Promotion activities on the part of the NCDF-municipalities themselves.
- Insertion of flyers in medical practices or pharmacies.
- Promotion activities by nursing homes and day care centers.

Advertisement channels of a regional scope mainly comprised regional newspapers. All NCDF-group leaders describe directly addressing to caregiving relatives as very important way to promote NCDFs, either by telephone contact or via Facebook or SMS.

Differences related to the variety and the spatial scope of the advertisement channels arose taking into account the spatial typology of the eponymous municipality. As far as the variety of information channels used is concerned, rural NCDF-municipalities stand out. Urban NCDF-municipalities were most imaginative with regard to directly addressing to caregiving relatives. Nevertheless, all NCDF-municipalities failed to reach LDCs. At this point, it should be noted that there is no information on how comprehensively contact building takes place and whether LDCs as a subgroup of caregiving relatives explicitly are contacted. This is in-line with a statement of a NCDF-group leader of a rural municipality that said the reason for failure is that LDCs do not know about the existence of NCDFs.

Nine further NCDF-group leaders referred to the non-attendance of LDCs and time constraints schedule clashes as the main reasons of LCDs not to be able to attend NCDFs (from urban, suburban and rural NCDFs-municipalities), and the fact that the caregiving relative cannot leave the cared-for person alone (mentioned by a rural NCDF-group leader).

37 of the 47 respondents explained why they could not answer this question. The main reasons were that on the one hand, NCDF-group leaders have too little information on this target group, and on the other hand, representatives of the group of LDCs do not participate in NCDFs.

### 4.2. Result 2: Knowledge on LDCs Strains and Assessment of LDCs’ Demand for NCDFs

The written expert survey brought into light that little is known about the strains of LDCs by NCDF-group leaders. Nevertheless, time constraints are assumed as the main obstacle for the demand for and participating in NCDFs.

Eleven out of 47 NCDF-group leaders addressed questions related to LDCs’ strains and burdens and presumed expectations related to NCDFs, and in this context, mainly referred to the challenge of overcoming extensive geographical distances between the places of residence of the LDC and the cared-for older parent(s). The answers of the respondents can be summarized to the following statements or conclusions:

- The necessity to overcome distances leads to time constraints and a state of emotional disorder (mentioned three times in total, including one urban and two rural NCDF-group leaders).
- Geographical distance creates uncertainty about the actual physical and psychological condition of the cared-for older parent(s) (mentioned two times in total, comprising one group leader each from a suburban and a rural NCDF-municipality).
- LDCs have more efforts with regard to the organization of care within the family [55] (two mentions in total, including one urban and one suburban NCDF-group leader).
- LDCs are plagued by ambivalent feelings [56]: on the one hand, geographical distance leads to an increase of emotional distance; on the other hand, geographical distance causes more worries (mentioned by a group leader of a rural NCDF-municipality).
- Travel expenses are a burden, especially in winter (mentioned by a group leader of a rural NCDF-municipality).
- One group leader of a rural NCDF-municipality is of the opinion that geographical distance furthermore makes it easier to talk about one’s (LDC-related) problems.
- Again, the reasons for the little information on this issue are that LCDs are out of the NCDF-group leaders’ sight.

### 4.3. Result 3: On the Potential of NCDFs as a Health Promotion Measure for LDCs

From the perspective of the NCDF-group leaders, NCDFs are thought to have a certain potential as a health promoting measure for LCDs. It significantly depends on the location of NCDFs if they will become a suitable and attractive offer for LDCs.

The following results are based on the information of 20 out of 47 NCDF-group leaders.

#### 4.3.1. Suggestions on Suitable Health Promotion or Support Measures for LDCs

The NCDF-group leaders’ suggestions relate to three different types of support measures:

1. Support measures that are linked with active involvement of LDCs. This includes the creation of a balanced care support mix, consisting of informal neighborhood assistance and professional care, mainly through mobile social and care services (mentioned three times in total, one from a group leader of an urban and two from group leaders of rural NCDFs); the willingness of LDCs to make use of existing offers (mentioned four times in total, all from group leaders of rural NCDFs); trying to organize care while the NCDF is taking place (mentioned by a group leader of a rural NCDF); the reduction of working time and claiming for more support from the social environment of the cared-for person (mentioned by a group leader from a rural NCDF).
2. Concessions from outside by taking into account the specific situation and strains of LDCs, e.g., in the context of medical appointments in order to save time (mentioned by two group leaders of each one suburban and rural NCDF).
3. The development of a particular telephone counseling offer (suggestions from two NCDF-group leaders, one suburban and one rural).

#### 4.3.2. Considerations on the Ideal Location for LDCs’ Nursing Discussion Forums

Under the condition that LDCs recognize NCDFs as an appropriate support measure—four out of 20 group leaders from all spatial types of NCDF-municipalities think so—we assume the importance of the right location to be a key factor for use.

While two group leaders of rural NCDFs, or rather nursing care discussion clusters, plead for locating NCDFs in the municipality where LDCs live, two further NCDF-group leaders (one from a suburban and one from a rural NCDF) suggest to establish NCDFs there, where “most of the efforts are”. Unfortunately, this statement is not explained in more detail.

## 5. Discussion

### 5.1. Appropriateness of Study Design, Representativenes and Reliability of Empirical Findings

Since LDCs are a neglected target-group in the public health sector in Austria [33,34], an explorative-qualitative case-study based approach, exploring the suitability of the current offer of NCDFs for LDCs in one of the nine provinces of Austria was chosen addressing the “providers’” perspective. For time exposure reasons, the geographical dispersion [57], of the NCDFs and due to the fact that there was no opportunity for third-party funding for this research, it was decided to conduct a written survey of all NCDF-group leaders in this province.

The results are based on a written survey of the NCDF-group leaders only show one side of the coin and call for research on the opinions of the LCDs relating to their support needs and ideas on the design of suitable NCDFs.

Nonetheless, related to the empirical findings a high representativeness is ensured due to a response rate of 61% out of all 77 Upper Austrian NCDFs. We can only speculate about the reasons for non-participation.

Given the fact that usually only one group leader is responsible for the NCDFs, in a way, the group leaders who have responded also reflect the situation in NDFs that, geographically speaking, have a regional dimension. It cannot be implied whether the results are true for the other eight Austrian provinces.

In addition, the limited information on LDCs indicates that the respondents know little about this target group and their health literacy and making use of health promotion offers. This, in turn, implies frankness in answering the questions and indicates reliability. Furthermore, the NCDF-group leaders cite the reasons for their knowledge deficit.

Since the NCDF-group leaders were not asked to unfold their assessment rules related to the life and strain situations of LDCs, it remains entirely open whether professional expertise and private experiences were mixed up.

However, it can be assumed that this is closely related to the fact that there still is a large knowledge gap on this target group in Austria [34].

### 5.2. Interrelations between the Profile and Advertisment of the Current NCDF-Offer and the Lack of Reachability of LDCs

At this point, first of all it should be noted that in general caregiving persons are less likely to use health-promoting measures compared to non-carers [9]. Referring to LDCs, one always has to keep in mind that time constrains are the focal obstacle that (could) hinder LDCs from making use of face-to-face and on-site health promotion offers. Besides that, the empirical results of the Upper Austrian case-study—in line with international findings [5,26]—show that a lack of information about availability of NCDFs [56] and the inappropriateness of the existing offer may explain the lack of reachability of LDCs.

Interestingly, the respondents did not interrelate reachability of LDCs with advertising efforts. This is quite comprehensible, as NCDFs are intensively advertised in urban and suburban, as well as in rural municipalities in a variety of ways, above all focusing the local level and directly addressing caregiving relatives—as was the case in 24 out of 47 cases—by telephone, Facebook or mail.

However, it must be recognized that the NCDF-group leaders neither have information on the procedure of the so-called “direct communication with LCDs”, nor are they able to assess the success of direct communication with the members of this specific target group.

### 5.3. On the Potential of NCDFs as a Health Promotion Measure and Requirements for a LDC-Suitable NCDF-Offer

Aside from the general problem of the measurability of the immediateness of effects of health-promotion measures [58], basing on the survey results, first of all it can be assumed that an ideal support, relief or health promoting measure never will exist due to the impossibility considering the great diversity of the LDCs’ living and caregiving situations [10]. At best, NCDFs will become a health promoting measure among others, above all web-based offers [36].

For the heterogeneous collective of LDCs, it is difficult to estimate the need for professionally-led support groups such as the NCDFs, because the caregiving situations, including the amount of formal, or rather professional (health and care), and of informal (future) family support on-site, as well as the degree of personally experienced strains associated to strategies differ from case to case. Considering this, sound empirical data on the use of health promotion measures by LDCs are still missing [25], but are urgently needed.

In addition, we still know little about the LDCs’ health literacy and self-awareness as a target group for health promotion, and in general. There is a knowledge gap on the meaning of professionally led on-site support groups for LDCs. Moreover, against the backdrop of time constraints, we do not know about whether LDCs give precedence to an informal chat with a friend with a cup of coffee over a professionally led conversation. It can be assumed that this decision also essentially depends on the content of the conversation (professional advice versus speaking something from one’s soul). Both in Austria and elsewhere, an in-depth discussion considering a wide-spread geographical scope on the issues of what is currently being discussed in NCDFs and—as far as LDCs are among the participants—what is burning in the soul of LDCs is needed.

Assuming that LDCs are or rather would be interested in face-to-face professionally led group-discussions on-site—regardless of whether the cared-for parent(s) is or are living at home or in a nursing home—based on the findings for the local caregiving relatives, only a small number of actual demanders can be expected. At this point, it is recalled that (1) the small number of participants of mainly female caregiving relatives (spouses) belonging to the first generation of caregiving relatives as well as the interrelation between the decline of use of health promotion measures and increasing age [4], (2) the small number of participants belonging to the second generation of caregiving relatives (daughters, daughters-in-law) as well as the observed declining trend of utilization and (3) the downwards trending NCDF-attendance [33].

Findings from the written survey referring to the differences between the strains and requirements of local caregiving relatives and LDCs and from literature can serve as a starting point for conceptualizing LDC-suitable NCDFs (see Table 7).

Although both target groups suffer from time constraints, physical distress dominates in local caregivers, whereas today’s LDCs are more likely to be psychologically overstrained [26,29,55]. It is to be expected that the psychological burdens of those LDCs, whose parent(s) in need of care are living in rural areas, will increase in the near future, because due to the continuing trend towards the beanpole family, the support potential within the family will decrease [35]. This, in turn, will raise particularly the loneliness among the very old [59]. Furthermore, in rural areas, the provision of daily goods and services, including mobile social and health care is thinning out and the accessibility of age-specific infrastructure facilities becomes more and more challenging [60,61,62]. These developments affect especially those LDCs who are on the one hand are single children, and whose spouses or partners experience the same caregiving situation on the other hand.

Since LDCs perceive geographical distances differently—on the one hand, they are experienced liberating and relieving, on the other hand, they can enhance the psychological pressure on LDCs—the perceived emotional pressure on LDCs also depends on their cared-for-older parents’ understanding for the demanding caregiving-situation and caregiving limitations [26]. Against this backdrop, the question arises of whether NCDFs for LDCs (in the future will) take over the function of “a safe haven” (according to justification and serving as time-outs from caregiving), focusing on exchanging personal caregiving experiences with others. Here, further healthcare research needs to focus more on health promotion measures from the perspective of the different target groups [10]. Due to the fact that non-carers are more likely to use health promotion measures than caregivers [9], and the assumption that work-life-balance becomes more important in society as well as it is predicted that health promotion will gain a higher social value in the future [63], referring to the potential of NCDFs as a health promotion measure, it is important to consider the following three questions:

1. Where should NCDFs be located in order to save time?
2. To what extent and at which locations could a handful of LDCs be brought together?
3. What are the requirements for tailor-made NCDFs in terms of organization and scope?

Based on the NCDF-group leaders’ assessments, the following conceptual relations can be derived (see Table 7), which need to be evaluated in the course of future quantitative-oriented studies exploring both the provider’s (NCDF-group leaders) and the “consumer’s” (LDCs’) perspective (see Table 7):

1. The profile of the NCDF-participants is as follows: women and men in midlife, of different marital status.
2. LDCs living in cities or towns with older parent(s) in need of care living in rural municipalities are not likely to make us of NCDFs located in their parents’ rural residential municipality. This may be due to the “fact” that LDCs only come for caregiving reasons and exclusively dedicate each minute to their parent(s). NCDFs are to be designed as a location-based offer in the residence municipalities or (urban) working places of LDCs.
3. Urban NCDFs might have a larger catchment area of LDCs and a larger number of (potentially interested) LDCs in comparison to NCDFs in rural municipalities.
4. At the moment, we can only speculate about the scope of tailor-made NCDFs for LDCs. Basing on evidence on the burdens and strains of LDCs, dealing with guilt and responsibility may be focal issues.
5. LDCs approach to NCDFS with different concerns in comparison to local caregivers. That is why it should be considered to offer target-group-specific and LDC-specialized NCDF-offers regardless of the spatial setting.

## 6. Conclusions

First of all, in view of the fact that we are still fishing in murky waters in many respects—among others due to the lack of statistical recording and the absence of other case-study based sound LDC-related empirical databases—and in order to limit the margin of interpretation, we must not be tempted to mix up factual data and private caregiving experiences when considering the suitability of NCDFs as a health promotion measure for the particular and heterogeneous target-group of LDCs. Referring to the results discussed above, the risk of overestimating of pithy single statements is substantial. Furthermore, at this time, there are no sufficient empirical findings in order to reflect the Austrian-wide situation, nor to judge the situation in other socioeconomically and socio-culturally comparable countries.

Moreover, LDCs are still an underexplored target group in the context of the perception and reception of health promotion measures such as NCDFs. Relating to the location planning of NCDFs, we have to admit that we still know little on the (future) profile of LDCs. A present, we only know where they are living or probably will live in the near future—their places of residence will further concentrate in urban municipalities (cities, small towns). However, what we do not know is whether and to what extent the next generation of LCDs will continue to feel obliged to care for their older parent(s) and whether their parent(s) will expect them to do so. For this reason, we do not know to what extent the NCDFs’ non-reachability of LDCs is or rather will remain a problem of fit or a problem of missing perception in terms of availability and receipt in terms of frequency [17].

That is why, in the future, the analysis of problems of fit—particularly related to NCDFs—must take into account: (a) the relevance of self-perception of LDCs as caregiving relatives, their feelings of belonging to a target-group, as well as their needs for emotional and informative support, (b) the state of knowledge and perceived attractiveness of different health promotion or support measures and (c) the (potential) competitive impact or complementary function of web-based, virtual support services and information platforms.

In a way this pilot study may serve as a starting-point for forming hypotheses on the suitability and future of NCDFs in the context of long-distance caregiving. What is needed here is another methodological approach, namely a quantitative investigation of LDCs, including their migration patterns, followed by an interdisciplinary interpretation of findings linked to demand and location planning of health promotion measures such as NCDFs.

On the assumption that professionally-led (group) support measures are or will always be very important to a small number of LDCs, too [11,38,48], NCDFs may experience a renaissance or generally gain importance as a health promotion measure. In order for this to work, public health needs to take the following steps:

1. Care and nursing professionals must be trained for the target-group of LDCs [34,55,57].
2. The target group of LDCs must be made visible to public. Thereby, NCDF-group leaders can act as multipliers.
3. LDCs must be given time to develop self-awareness as a target group of health promotion measures. For that reason, NCDFs can serve as on-site platforms for the development of mindfulness, health literacy and community building. In turn, all three are important prerequisites for making use of health promotion measures [64].
4. Pilot projects (“NCDFs for LDCs”) should be initiated in different spatial settings and accordingly advertised in order to make LDCs aware of this kind of support or health promotion offer [58]. As a first step, it would be interesting to explore what (currently) is being debated in the NCDFs of other provinces of Austria, in order to identify the support needs of LDCs and to be able to sharpen ideas on the demand, organization and scope of (future) suitable offers. It should to be kept in mind that NCDFs cannot eliminate distress and burdens that directly are related to overcoming extensive geographical distances, but nevertheless may improve quality of life [60].
5. After running for a reasonable period, NCDFs need to be evaluated and linked to basic research on intervention programs for informal caregivers [55]. Only then it will become apparent whether NCDFs may be a suitable health promotion measure for LDCs and may exist alongside web-based offers.

## Figures and Tables

**Table 1:** Structure and content of the questionnaire for the written expert survey.

Structure of Questionnaire	Subjects
thematic block no. 1: demographic profile of NCDF-group leaders	sex age (two closed questions)
thematic block no. 2: organization and catchment area of NCDFs	name of eponymous and partner municipalities location day of the week, time, duration, frequency of place-taking catchment areas related to attendees changes in demand advertising cooperation with other municipalities (nursing care discussion cluster) (11 open-ended questions)
thematic block no. 3: demographic profile of participants places of residence of participating caregiving relative and cared-for person	sex age number of participants family relationship between attendee and cared-for person places of residence of NCDF-participant and cared-for person (3 closed and 2 open-ended questions)
thematic block no. 4: NCDF-group leaders‘ assessment of the NCDFs‘ health-promoting effects	expectations of participants strains, burdens and concrete concerns of participants explicit contribution on the participants’ health and well-being (4 closed questions)
thematic block no. 5: knowledge on burdens related to long-distance caregiving and considerations related to target-group-specific support and relief measures (including NCDFs)	challenges and concerns related to extensive commuting distances considerations on the reachability of long-distance caregiving relatives by NCDFs considerations on the characteristics of ideal support and relief measures for long-distance caregivers considerations on the relevance and location planning of NCDFs (3 open-ended questions)

**Table 2:** Structure and content of the questionnaire for the written expert survey.

Spatial Typology of Upper Austria’s NCDF-Municipalities	In Total	Covered in the Written Survey [%]
urban centers	10	5 [50%]
regional centers	2	0 [0%]
suburban municipalities	20	15 [75%]
rural municipalities	45	27 [60%]
in total	77	47 [61%]

**Table 3:** Breakdown of basic information on NCDFs.

Basic Information on Group Leaders and Organization of NCDFs	In Urban Centers	In Suburban Municipalities	In Rural Municipalities
age of group leaders	aged 43–58	aged 33–62	aged 42–68
sex of group leaders	entirely women	entirely women	mainly women (24 out of 27)
sharing their function as group leaders	mainly yes (4 out of 5)	no	mainly not (22 out of 27)
frequency of place-taking	mostly once a month (4 out of 5) in one case: twice a month	mostly once a month (12 out of 15) in one case: twice a month in two further cases: bimonthly	mostly once a month (26 out of 27) in one case: twice a month
day of the week	during the week (Mon, Tue or Thu)	during the week (Mon, Tue, Wed or Thu)	during the week (Mon, Tue, Wed or Thu)
start time	between 6 and 7 p.m.	mostly between 7 and 8 p.m. (13 out of 15) in one case: 2 p.m. in one further case: between 5 and 7 p.m.	between 7 and 8 p.m.
duration	2 h	0.5–2 h	2–2.5 h

**Table 4:** Theoretical and actual catchment areas of NCDFs (eponymous and nursing care discussion clusters) related to the number of domestically cared-for older persons living in NCDF location municipalities.

NCDFs’ Catchment Area	Spatial Type of NCDF Eponymous Municipality	Number of NCDFs	Theoretical Catchment Area of NCDFs Related to Domestic Cared-for Older Persons *)	Actual Catchment Area of NCDFs Related to Domestic Cared-for Older Persons *)	Relation between Theoretical and Actual Catchment Area [in %]
limited to eponymous municipality	urban centers suburban municipalities rural municipalities	4 5 9	9300 500 1300	9300 500 1300	100% 100% 100%
comprising partner municipalities	urban centers suburban municipalities rural municipalities	1 10 18	200 2600 4700	<100 1800 2100	10% 69% 44%
related to all NDFs	urban centers suburban municipalities rural municipalities	5 15 27	9500 3100 6100	9200 2300 3400	98% 74% 56%
in total		47	18,700	15,000	80%

* Values rounded to 100s.

**Table 5:** Profile of NCDF-participants belonging to the second generation of caregiving relatives.

Profile of Participants	NCDFs in Urban Centers	NCDFs in Suburban Municipalities	NCDFs in Rural Municipalities
participants per NCDF	4–5 (5 out of 5)	4–8 (15 out of 15)	3–15 (25 out of 27)
development of NCDF-attendance	trending downwards (3 out of 5)	trending downwards (8 out of 15)	trending downwards (12 out of 27)
age of participants	aged 50–60 (5 out of 5)	aged 50–70 (15 out of 15)	aged 45–70 (27 out of 27)
sex of participants	mainly women (4 out of 5)	mainly women (13 out of 15)	mainly women (24 out of 27)
relationship of places of residence of participants and their cared-for older relative/s	mostly living in the same municipality (3 out of 5)	mostly living in the same municipality (11 out of 15)	mostly living in the same municipality (22 out of 27)
kinship of participants and older cared-for person/s	daughter > daughter-in-law (5 out of 5)	daughter > daughter-in-law (15 out of 15)	daughter > daughter-in-law (27 out of 27)
older cared for person/s	mother > father > parent-in-law (5 out of 5)	mother > parent-in-law > father (15 out of 15)	mother > mother-in-law > father/father-in-law (27 out of 27)

**Table 6:** Advertising NCDFs.

	NCDFs in Urban Centers	NCDFs in Suburban Municipalities	NCDFs in Rural Municipalities	Number of Advertisement Channels in Total
being advertised (information provided: 44 out of 47 NCDF-group leaders)	yes: 5 out of 5	yes: 14 no, no longer: 1	yes: 25 no, no longer: 2	
most relevant advertisement channels of local scope (number of channels)	organized by municipality (municipal newspapers, homepage) > leaflets (community physician, pharmacy) > information provided by hospital (5 cases)	municipal newspapers > community physician/pharmacy > announcements of the church (10 cases)	community physician/pharmacy > nursing homes/day-care centers for elderly people > leaflets of the municipality > announcements of the church (10 cases)	11 in total
most relevant promotion channels of regional scope (number of channels)	regional newspapers (1 case)	regional newspapers > health fairs/health days (3 cases)	regional newspapers > announcements of the initiative “healthy communities” (4 cases)	4 in total
directly contacting caregiving relatives (number of channels)	personal acquaintance/telephone contact, written invitation, word-of-mouth advertising (3 cases)	personal acquaintance/telephone contact, written invitation, word-of-mouth advertising (1 case)	personal acquaintance/telephone contact, written invitation, word-of-mouth advertising > SMS, Facebook, Whatsapp (2 cases)	4 in total
most relevant promotion channels not specified scope (number of channels)	none	posters, events (1 case)	posters, events (1 case)	1 in total
ranking of promotion channels related to relevance	information with local scope > direct contacts to caregiving relatives > information channels of regional scope	information with local scope > information channels of regional scope > direct contacts to caregiving relatives	information with local scope > direct contacts to caregiving relatives > information channels of regional scope	

**Table 7:** Considerations on the requirements for LDCs’ and local caregiving relatives’ suitable NCDFs.

Information Derived from Expert Survey and Literature	NCDFs for Long-Distance Caregiving Relatives	NCDFs for Local Caregiving Relatives
current demand of NCDFs	still unknown	to a limited extent
relevance and further (quantitative) development of the target group	increasing	increasing
further development of psychological burdens	rising	rising
demand for professionally led-discussion groups	still unknown	to a limited extent
relevance of web-based alternative services	probably relevant	increasing
future demand for NCDFs	still unknown	unknown or in decline
appropriate name of offer	“Supervised Discussion Forums for LDCs”	“Regular’s Tables for Caregiving Relatives”
assumed motivation for participation	mutual exchange with similarly affected caregiving relatives supervised speaking-from-the-soul getting in touch with health and care professionals staying informed about support measures and developments in domestic care	time-outs / short breaks from domestic care gaining personal distance exchange with similarly affected caregiving relatives information and counseling
demographic profile of (future) NCDF-participants	women (and men) caregiving relatives of the second generation (particularly daughters/sons)	women (and men) caregiving relatives of the first and second generation
relation LDCs/local caregivers	solely LDCs	solely local caregiving relatives
assumed number of participants per NCDF	a couple of people	as at present
regularity of participation [57]	useful, but still unknown	useful, but still unknown
appropriate advertising channels	word-of-mouth advertising information (leaflets) in medical practices directly being contacted by care and nursing professionals information channels with super-regional scope (e.g., newspapers, internet platforms)	as at present
catchment area	local (residential municipalities of LDCs)	local (residential municipalities of local caregiving relatives) or small-scaled (neighbor municipalities)
appropriate location municipality for NCDFs	residential municipality of the LDC: mostly in the urban or suburban	residential municipality of the local caregiving relative: urban, suburban, as well as rural
location/meeting place	at the workplace attractive meeting places (e.g., cafes)	attractive meeting points (cafes)
organization structure depending on location	on demand, during the week during lunch breaks, in the afternoon and (early) evening	on demand, during the week, in the (early) evening
assumed scope of NCDFs (scope/discussed issues)	constructing identity as caregivers [55] developing mindfulness coping with guilt and feelings of obligation and responsibility	dealing with “unlimited” availability
relevance and function of care and nursing expert (=NCDF-group leader)	Informant, counselor mediator trainer	link between oneself and the cared-for older parent(s)
